# Population genetics and evolutionary history of the endangered Eld’s deer (*Rucervus eldii*) with implications for planning species recovery

**DOI:** 10.1038/s41598-021-82183-7

**Published:** 2021-01-28

**Authors:** Mirza Ghazanfarullah Ghazi, Surya Prasad Sharma, Chongpi Tuboi, Sangeeta Angom, Tennison Gurumayum, Parag Nigam, Syed Ainul Hussain

**Affiliations:** Wildlife Institute of India, Chandrabani, Post Box #18, Dehra Dun, Uttarakhand 248002 India

**Keywords:** Computational biology and bioinformatics, Ecology, Evolution, Genetics, Zoology

## Abstract

Eld's deer (*Rucervus eldii*) with three recognised subspecies (*R. e. eldii, R. e. thamin,* and *R. e. siamensis*) represents one of the most threatened cervids found in Southeast Asia. The species has experienced considerable range contractions and local extinctions owing to habitat loss and fragmentation, hunting, and illegal trade across its distribution range over the last century. Understanding the patterns of genetic variation is crucial for planning effective conservation strategies. This study investigated the phylogeography, divergence events and systematics of Eld's deer subspecies using the largest mtDNA dataset compiled to date. We also analysed the genetic structure and demographic history of *R. e. eldii* using 19 microsatellite markers. Our results showed that *R. e. siamensis* exhibits two divergent mtDNA lineages (mainland and Hainan Island), which diverged around 0.2 Mya (95% HPD 0.1–0.2), possibly driven by the fluctuating sea levels of the Early Holocene period. The divergence between *R. e. eldii* and *R. e. siamensis* occurred around 0.4 Mya (95% HPD 0.3–0.5), potentially associated with the adaptations to warm and humid climate with open grassland vegetation that predominated the region. Furthermore, *R. e. eldii* exhibits low levels of genetic diversity and small contemporary effective population size (median = 7, 4.7–10.8 at 95% CI) with widespread historical genetic bottlenecks which accentuates its vulnerability to inbreeding and extinction. Based on the observed significant evolutionary and systematic distance between Eld’s deer and other species of the genus *Rucervus,* we propose to classify Eld's deer (*Cervus eldii*) in the genus *Cervus*, which is in congruent with previous phylogenetic studies. This study provides important conservation implications required to direct the ongoing population recovery programs and planning future conservation strategies.

## Introduction

Small populations are susceptible to loss of genetic diversity as a consequence of founder events, genetic bottlenecks, and inbreeding, accelerated by the cumulative impacts of natural and anthropogenic factors^1^. Species characterised by fragmented and isolated populations are increasingly prone to factors that can lead to reduced levels of genetic diversity and loss of evolutionary potential which, in turn, limit the populations' ability to adapt to the changing environment^2,3^ and as a consequence, elevate the risk of local extinctions^4,5^. Therefore, preservation and maintenance of adequate amount of genetic diversity in natural populations of threatened species is crucial for their long-term survival in the wild^6^.

**Figure 1:**
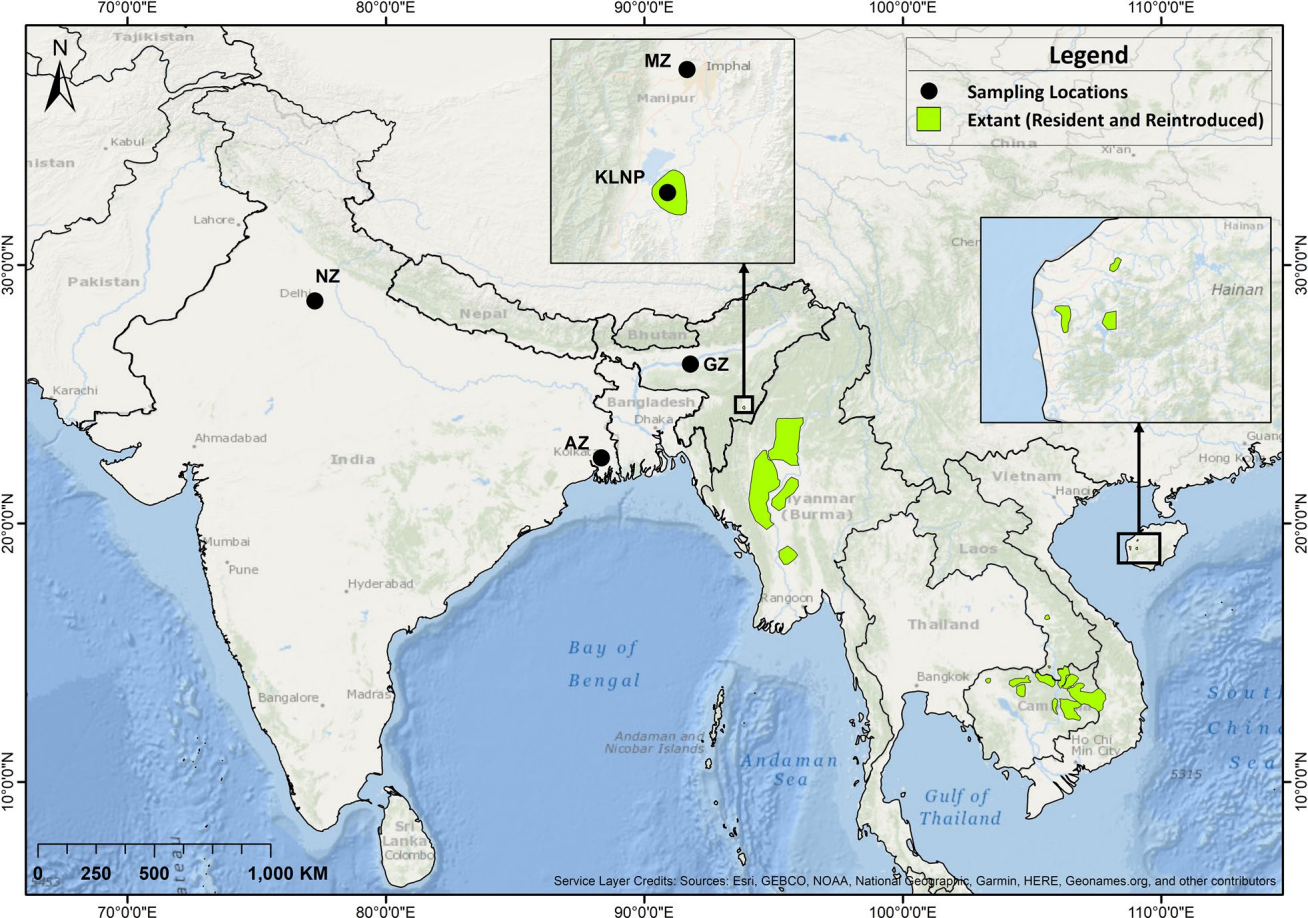
Map showing the geographical distribution of Eld’s deer (*Rucervus eldii*) obtained from the IUCN Red List^7^. Sampling locations are represented as *NZ* National Zoological Park, New Delhi; *AZ* Alipore Zoological Garden, West Bengal; *GZ* Assam State Zoo, Assam; *MZ* Manipur Zoological Garden, Manipur; *KLNP* Keibul Lamjao National Park, Manipur. The map was created using ArcGIS v.10.3.1 software developed by ESRI (https://www.esri.com).

Eld's deer or brow-antlered deer (*Rucervus eldii*) is a localised and endangered tropical Southeast Asian cervid^7^. It was once widely distributed across South and Southeast Asia, extending from Manipur in Northeast India to Myanmar, Thailand, Cambodia, Vietnam up to Hainan Island in China^8,9^. Over the past 200 years of known history, the species has witnessed severe range contraction across its historical distribution range. Overexploitation of natural resources, habitat loss and fragmentation, hunting, and illegal trade are the major cause of its population decline^7,10^. The species at present is listed as endangered by the IUCN Red List^7^. It is classified into three subspecies; Manipur's brow-antlered deer (*R. e. eldii*), Burmese brow-antlered deer (*R. e. thamin*), and Siamese Eld's deer *(R. e. siamensis*)^7^. However, the Hainan Island (HI) population of *R. e. siamensis* is, at times, considered to be the fourth subspecies of Eld's deer^11,12^. The current distribution of Eld's deer subspecies is highly fragmented (Fig. 1), with several regional exterminations from its former distribution range^7^. The Manipur's brow-antlered deer (*R. e. eldii*) was declared extinct in 1951, and the current wild population in Keibul Lamjao National Park (KLNP) in Manipur, India has recovered from 14 individuals rediscovered in 1975^13^. It represents the most threatened subspecies of Eld’s deer which is highly susceptible to local extinction compared to *R. e. thamin* and *R. e. siamensis*, due to its small population size and restricted habitat in KLNP^14^. Despite its vulnerability to inbreeding and extinction due to stochastic and demographic factors, limited information is available on the genetic status of this subspecies to assist its population management programmes. *R. e. siamensis* underwent a significant decline across its distribution range during the last century and was declared extinct from Thailand and Vietnam. Presently, the subspecies occurs in scattered populations in Lao PDR and Cambodia and Hainan Island in China^7^. *R. e. thamin* has also witnessed severe range contractions and is considered extinct from Thailand. Presently, this subspecies represents the largest population of Eld’s deer in the wild with localised populations in Myanmar and introduced populations in Thailand^7,15^.

Thailand is considered as the historical centre of the geographical distribution range of Eld’s deer^16^. It is also an area of biogeographical importance as it facilitated the historical migration of large mammals between South China and Java. The oldest fossil record of Eld’s deer is known from the Quaternary reddish clay deposits of Middle Pleistocene in north-eastern Thailand^16,17^. Another fossil form of Eld’s deer was discovered in the Island of Java, Indonesia, dating back to around 3000 years before present (Ybp)^18^ indicating the historical distribution of Eld’s deer in this region which is presently isolated due to the rise in eustatic sea levels. Evidence of faunal compressions and local extinctions due to the increased sea levels has been reported in the Isthmus of Kra region, which divides the Indochinese and Sundaic sub-regions^18,19^. These changes together with dynamic climatological and vegetational conditions facilitated the diversification of many ruminant species^20–23^ and would have influenced the patterns of genetic diversity, divergence and evolutionary history of Eld’s deer subspecies.

A taxonomic conundrum of assigning Eld's deer to either genus *Cervus* or *Rucervus* still persists. Eld's deer share morphological similarities with swamp deer (*Rucervus duvaucelii*) and the extinct Schomburgk's deer (*R. schomburgki*)^24^*.* Based on these affinities, all three species are currently classified together in the genus *Rucervus*^24,25^. However, previous molecular studies assigned Eld’s deer to the genus *Cervus*^26–29^ but Grubb^8^ revived Thomas’s^30^ classification and assigned the species to genus *Rucervus* which is currently adopted by the IUCN Species Survival Commission. Despite the species being globally endangered, few studies have attempted to assess its genetic status^12,29,31^. Studies on microsatellite genetic variation are limited to the HI population of *R. e. siamensis*^11^. Lack of adequate information on the spatial genetic structure and levels of genetic variability among different populations of Eld’s deer subspecies is an impediment for planning effective conservation breeding and population management strategies.

In this study, we integrated the available mitochondrial DNA (mtDNA) control region dataset to assess the patterns of genetic diversity, phylogeography and phylogenetic relationships among the Eld's deer subspecies. We analysed the complete mitochondrial genomes of *R. e. eldii* and *R. e. siamensis* to estimate the age of these subspecies and the major divergence events within the Eld's deer group. To resolve the taxonomic dilemma of assigning Eld's deer to either genus *Rucevus* or *Cervus*, we reconstructed the phylogenetic association of species of tribe *Cervini* using Bayesian analysis of complete mitochondrial genomes. We also estimated the microsatellite genetic diversity, contemporary and ancestral effective population size (*N*_*e*_), and demographic history of *R. e. eldii* using microsatellite markers. Due to logistical and legal constraints of range-wide biological sampling of Eld’s deer subspecies, we confined the microsatellite analyses to *R. e. eldii.* The findings of this study will provide insights into the patterns of genetic association among the subspecies with a deeper understanding of the evolutionary history of Eld's deer, which is essential for planning conservation and management strategies.

## Results

### Phylogeography and genetic differentiation

**Figure 2:**
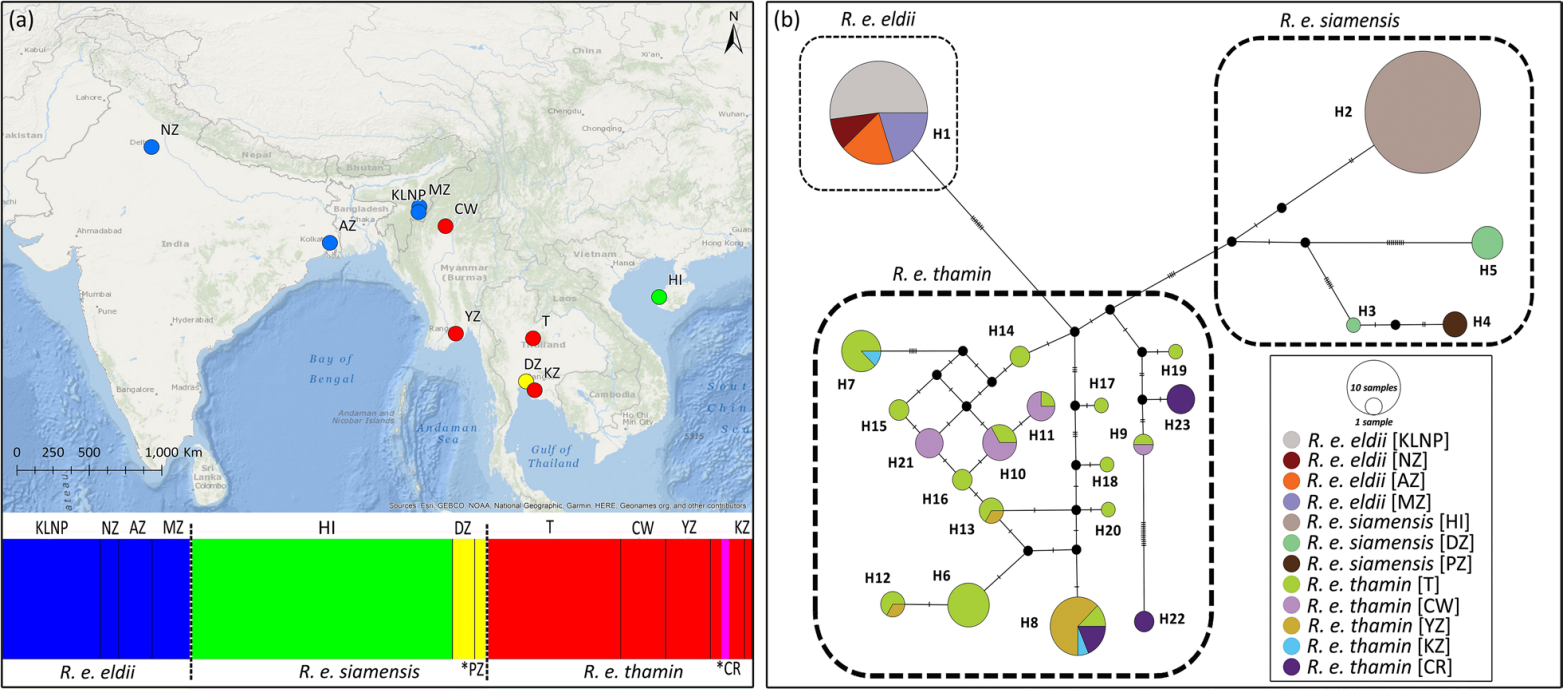
(**a**) Bayesian analysis of population structure (BAPS) of Eld’s deer (*Rucervus eldii*) subspecies identified four genetic clusters using 200 mitochondrial control region sequences. Clusters are represented by colours. The dashed black lines separate subspecies and solid black lines separate populations. The map was created using ArcGIS v.10.3.1 software developed by ESRI (https://www.esri.com). (**b**) Median-joining haplotype network for 23 control region haplotypes identified among the three Eld’s deer subspecies. Three major clusters were recovered and are indicated by subspecies names. Circle size represents the frequency of individuals, and colour defines the populations. Small black circles represent median vectors. Mutational steps are shown by bar on the respective branches. The populations are abbreviated as *KLNP* Keibul Lamjao National Park, Manipur; *NZ* National Zoological Park, New Delhi; *AZ* Alipore Zoological Garden, West Bengal; *MZ* Manipur Zoological Garden, Manipur; *HI* Hainan Island, China; *DZ* Dusit Zoo, Thailand; *PZ Paris Zoo, France (not shown in map); *T* Thailand; *CW* Chatthin Wildlife Sanctuary, Myanmar; *YZ* Yangon Zoo, Myanmar; *KZ* Khao Khew Open Zoo, Thailand; *CR Conservation & Research Centre, USA (not shown in map).

A total of 23 haplotypes were observed in the control region (447 bp) of three subspecies of Eld’s deer segregated by 126 polymorphic sites with an overall haplotype diversity; (Mean ± SD) *hd* = 0.805 ± 0.020 and low nucleotide diversity; *Pi* = 0.023 ± 0.0005. All the three subspecies of Eld’s deer contain unique control region haplotypes localized within the populations of the respective subspecies (Fig. 2b). The captive and wild populations (n = 71) of *R. e. thamin* contain 18 haplotypes (*hd* = 0.908 ± 0.018) out of the total 23 haplotypes observed in all the three subspecies. *R. e. siamensis* exhibited four control region haplotypes (n = 9) with *hd* = 0.639 ± 0.126. Interestingly, we observed no haplotype sharing among the populations of *R. e. siamensis*. All the identified haplotypes in *R. e. siamensis* were unique to their respective populations with one haplotype (H2) observed in the Hainan Island, two haplotypes (H3 and H5) in Dusit Zoo, Thailand, and one haplotype (H4) in Paris Zoo, France. All the unique individuals of the captive (n = 23) and wild populations of *R. e. eldii* (n = 27) identified using 19 microsatellite loci shared a single control region haplotype.

**Figure 3:**
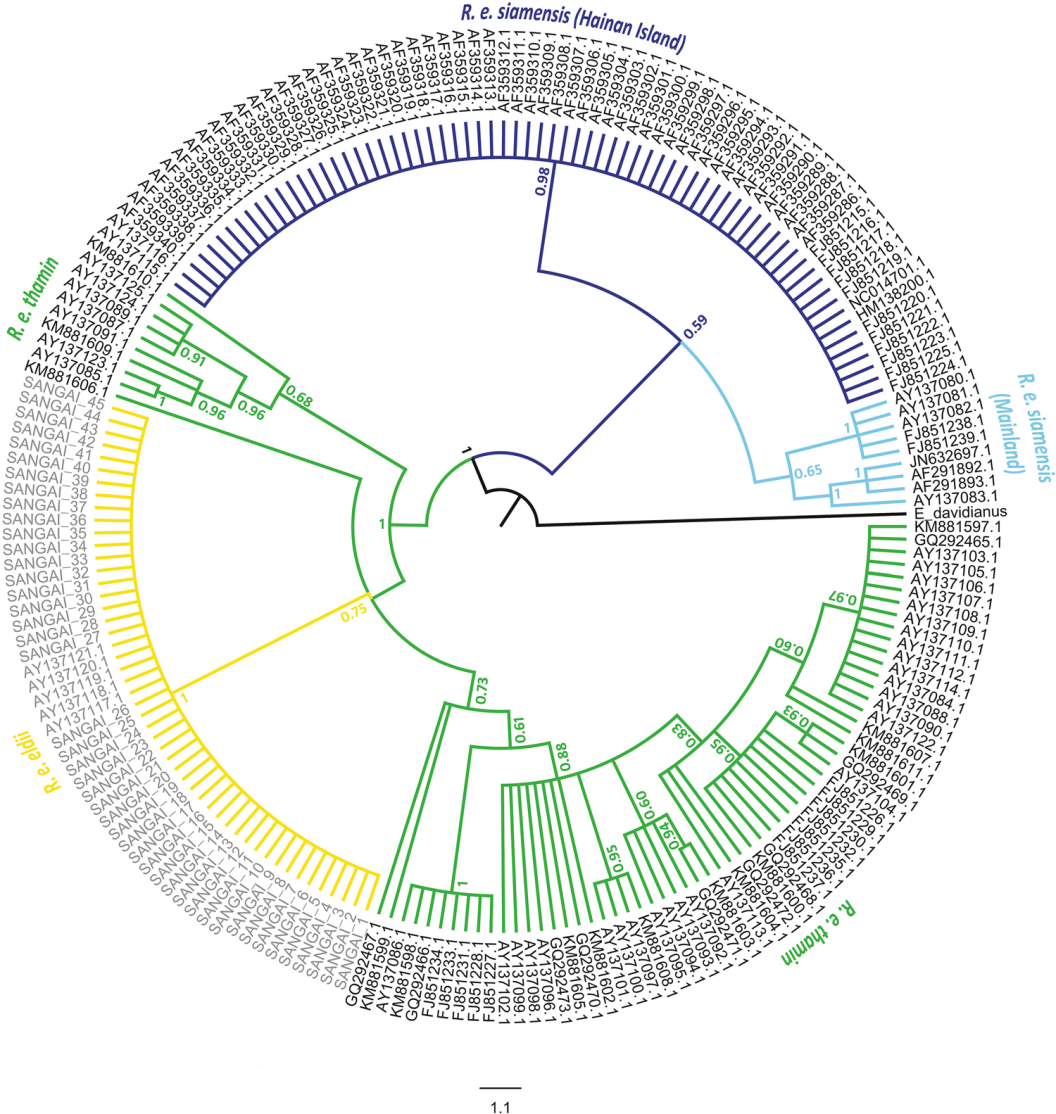
Phylogenetic tree of three Eld's deer (*Rucervus eldii*) subspecies obtained from the Bayesian analysis of mitochondrial control region sequences (447 bp). Bayesian Posterior Probability (BPP) values are provided at respective nodes.

The median-joining network revealed three haplotype clusters among the Eld's deer subspecies (Fig. 2b). *R. e. eldii* was separated from *R. e. thamin* and *R. e. siamensis* by a minimum of 9 and 16 mutational steps, respectively. Moreover, the haplotype network differentiated the mainland and HI populations of *R. e. siamensis* by ten mutational steps. In comparison, mainland populations of *R. e. siamensis* were separated by 13 mutational steps from *R. e. thamin*. Furthermore, the HI population showed a sequence variation of ~ 2.7% with both the mainland populations of *R. e. siamensis* and *R. e. thamin* at the control region (see Supplementary Table S1 online). Bayesian Analysis of Population Structure (BAPS) based on non-spatial clustering with admixture analysis using control region sequences recovered four major genetic clusters among the Eld’s deer subspecies (Fig. 2a). Interestingly, the mainland and HI populations of *R. e. siamensis* were segregated into two genetic clusters. The identified clusters were significantly differentiated with Fst values ranging from 0.347 to 1.00 (see Supplementary Table S1 online). Phylogenetic analysis of the control region based on the Bayesian approach recovered two statistically supported clades and recovered non-monophyletic relationships among Eld's deer subspecies (Fig. 3). The first clade of *R. e. siamensis* comprised of two sub-clades with mainland and HI populations nested as sister groups. The second clade suggested an ambiguous association as *R. e. eldii* was nested within the *R. e. thamin* populations.

### Divergence times and phylogenetic analyses

Sequencing of the complete mitochondrial genome of *R. e. eldii* obtained a sequence of 16,357 bp comprised of 13 Protein Coding Genes (PCGs), 22 tRNA genes, two rRNA genes, and a non-coding control region (see Supplementary Fig. S1 online). All the three subspecies of Eld's deer exhibit unique control region haplotypes with no haplotype sharing across the subspecies. Subsequently, the mitogenomes obtained from the GenBank were assigned to their respective subspecies based on the geographic range and signatures of the mtDNA control region. We identified all the three sequences obtained from GenBank to be of *R. e. siamensis* (Accession No. JN632697, NC014701 and HM138200). Further, the HI and mainland populations of *R. e. siamensis* also exhibited unique control region haplotypes. Therefore, we assigned two mitogenomes to the HI population (NC014701 and HM138200) and one to the mainland population based on the similarity indices of the blast search and estimated the divergence between these populations of *R. e. siamensis*. The obtained value for the index of substitution saturation (*Iss*)^32^ for the analysed mitogenome dataset was 0.4093 and the critical *Iss.c* values for the symmetrical and asymmetrical trees were 0.8424 (*P* < 0.001) and 0.6743 (*P* < 0.001), respectively. Both the values of *Iss.c* were significantly higher than the observed *Iss* values. Therefore, the complete mitogenome dataset was found suitable for phylogenetic and divergence analysis.

### Divergence times

**Figure 4:**
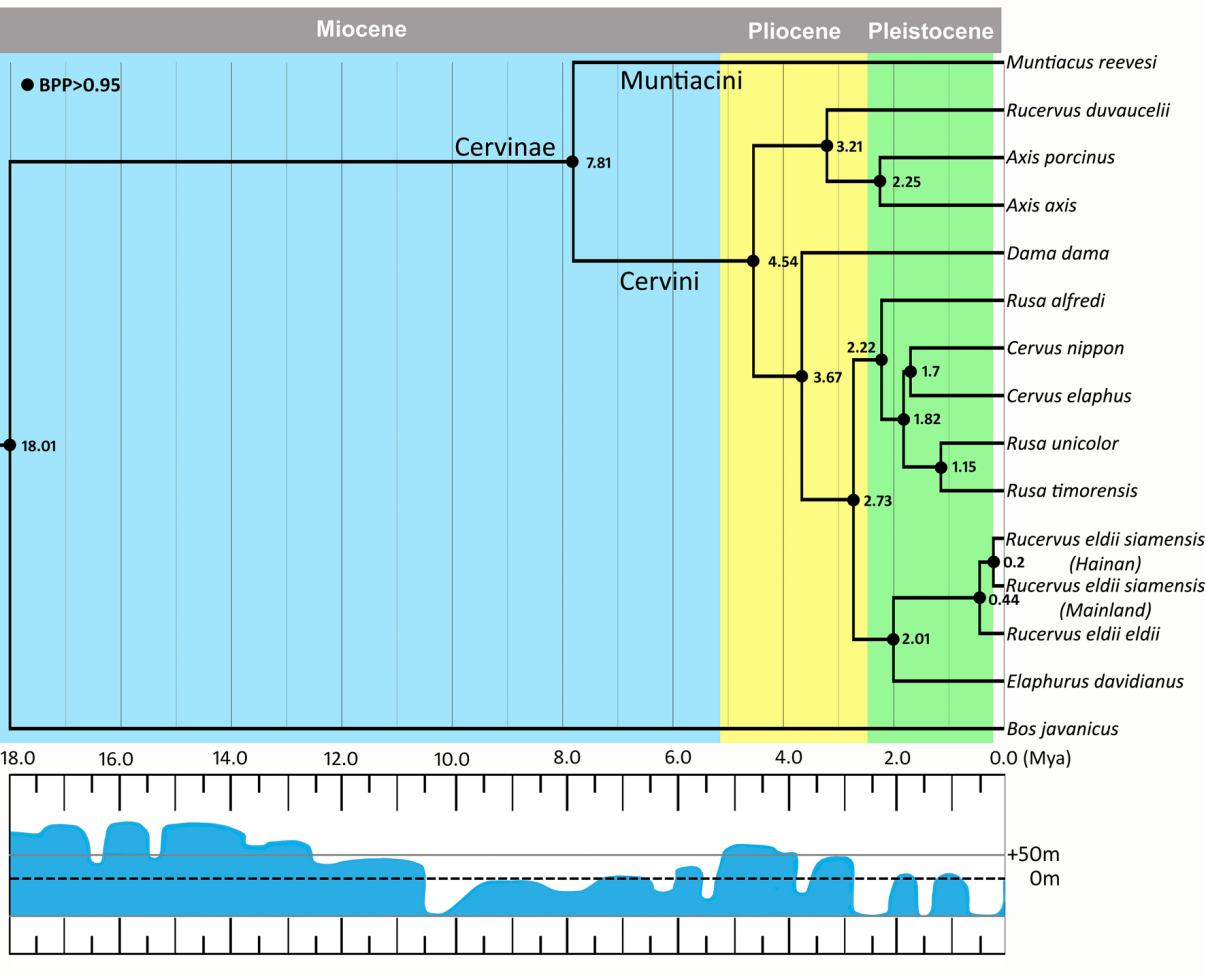
Time-calibrated phylogenetic tree of the tribe *Cervini* extending from 18 Mya to present based on Bayesian analysis of mitochondrial genomes. Approximate estimates of the eustatic sea level changes are presented under the chronogram^35^. The current sea levels are represented by the dashed line (0 m). The geological time scale was acquired from the Geological Society of America.

The divergence time estimation based on Bayesian analysis of 13 PCGs, two rRNA genes (12S and 16S), and control region sequences obtained a strongly supported time-calibrated tree, which is consistent with the previous phylogenetic studies^26,27^ (Fig. 4). Approximations of time to the most recent common ancestor (T_MRCA_) with 95% Highest Posterior Densities (HPD) is provided in Supplementary Table S2 online. The details of parameter convergence of divergence analyses are provided in Supplementary Fig. S2 online. The estimates of molecular divergence revealed simultaneous diversification of two evolutionary lineages within the tribe *Cervini*. We recovered an early divergence of common ancestors of the genera *Axis* and *Rucervus* ~ 4.5 million years ago (Mya) (95% HPD 3.6–5.4) from the main phylogenetic stock of tribe *Cervini*. The split between Eld's deer and the *Cervus-Rusa* group occurred around 2.7 Mya (95% HPD 2.1–3.3) during the Late Pliocene. Subsequently, the diversification of *Cervus* and *Rusa* species took place around 1.8 Mya (95% HPD 1.4–2.2). However, early separation of Philippine spotted deer (*Rusa alfredi*) from the common ancestors of the *Cervus/Rusa* group around 2.2 Mya (95% HPD 1.7–2.7) indicates that *R. alfredi* is the oldest form of living rusine deer.

Simultaneously, the divergence of *Elaphurus davidianus* and *R. eldii* took place ~ 2.0 Mya (95% HPD 1.5–2.5) during the Early Pleistocene period. Within *R. eldii*, we obtained two diversification events. The divergence of *R. e. eldii* from *R. e. siamensis* which occurred ~ 0.4 Mya (95% HPD 0.3–0.5), followed by the divergence of HI and the mainland populations of *R. e. siamensis* ~ 0.2 Mya (95% HPD 0.1–0.2).

### Phylogenetic analysis

Phylogenetic reconstruction of tribe *Cervini* based on 13 PCGs, two rRNA genes and control region sequences using the Bayesian approach yielded a statistically supported phylogenetic tree of tribe *Cervini* (see Supplementary Fig. S3 online). *Bos javanicus* was nested at the root of the phylogenetic tree as an out-group. Within tribe *Cervini*, we recovered the genera *Axis, Cervus*, and *Dama* to be monophyletic (Bayesian Posterior Probability, BPP = 1). Fallow deer (*Dama dama*) and Persian fallow deer (*Dama mesopotamica*)*,* chital (*Axis axis*) and hog deer (*Axis porcinus*) were allied together as sister species in their respective clades. Within the genus *Cervus*, all three species; red deer (*Cervus elaphus*)*,* sika deer (*Cervus nippon*) and white-lipped deer (*Cervus albirostris*) were monophyletic (BPP = 1). However, the genus *Rucervus* and *Rusa* were observed to be paraphyletic. *R. duvaucelii* was nested with the genus *Axis* (BPP = 1) while *R. eldii* was associated with Pére David's deer (*Elaphurus davidianus*) as a sister group. The monophyly of genus *Rusa* was disrupted by the disjunct position of *R. alfredi*, which was allied to the clade comprised of species of the genus *Cervus* and *Rusa* (*R unicolor/R. timorensis*).

### Microsatellite genetic variation in *R. e. eldii*

We successfully genotyped 66 out of total 79 faecal pellet samples, with 48 samples from KLNP and 18 samples from captive populations including Manipur Zoological Garden (n = 6), Assam State Zoo (n = 8) and National Zoological Garden (n = 4). The remaining samples from Manipur Zoological Garden (n = 4) and Alipore Zoological Garden (n = 9) failed to amplify, therefore excluded from the microsatellite analysis (see Supplementary Table S3 online). Based on multi-locus match, we identified 24 unique individuals out of 48 samples genotyped from KLNP and 18 individuals from 18 samples from captive populations. Therefore, a total of 42 samples (Wild = 24 and Captive = 18) were further used to perform population genetic analyses. We obtained low levels of microsatellite genetic variation in captive populations (Mean ± SE) (*H*_*o*_ = 0.13 ± 0.04; *H*_*e*_ = 0.31 ± 0.05) compared to the wild population of *R. e. eldii* (*H*_*o*_ = 0.31 ± 0.05; *H*_*e*_ = 0.47 ± 0.03) in KLNP (see Supplementary Table S4 online). The mean allelic richness for the wild population was 2.68 ± 0.15 and for the captive population was 2.11 ± 0.21. All the 19 microsatellite loci were polymorphic for the wild population of KLNP and obtained a total of 51 alleles. However, four loci (RT1, DF/R, BM4107, and AF232760) were monomorphic in the captive populations with a total of 40 alleles observed across 19 microsatellite loci. Most of the loci showed significant deviation from the Hardy–Weinberg equilibrium (HWE), which can be attributed to heterozygosity excess and inbreeding. The Bayesian clustering analysis using STRUCTURE revealed the presence of two genetic clusters based on Δ*K* statistics (see Supplementary Fig. S4 online). The KLNP individuals were assigned to the first cluster whereas all the individuals of captive origin were assigned to the second cluster (see Supplementary Fig. S5 online). The genetic differentiation estimates between the captive and wild populations of *R. e. eldii* obtained low to moderate levels of differentiation based on the fixation measures (Fst = 0.26; *P* < 0.001); Gst = 0.15 (*P* < 0.001) and allelic differentiation (Jost’s D = 0.12; *P* < 0.001). The estimates of the inbreeding coefficient (*F*) were 0.342 ± 0.100 and 0.566 ± 0.098 in the wild and captive populations, respectively.

### Past population changes in *R. e. eldii*

The bottleneck detection using heterozygosity excess showed strong signature of genetic bottleneck. The one-tailed Wilcoxon test for heterozygosity excess was significant for all the three mutation models (*P* < 0.001), viz. Infinite Allele Model (IAM); Two-phase Model (TPM) and Stepwise Mutation Model (SMM) (see Supplementary Table S5 online). The allelic frequency distribution also recovered a mode-shift curve, which confirms that the wild population of *R. e. eldii* has gone through a genetic bottleneck.

**Figure 5:**
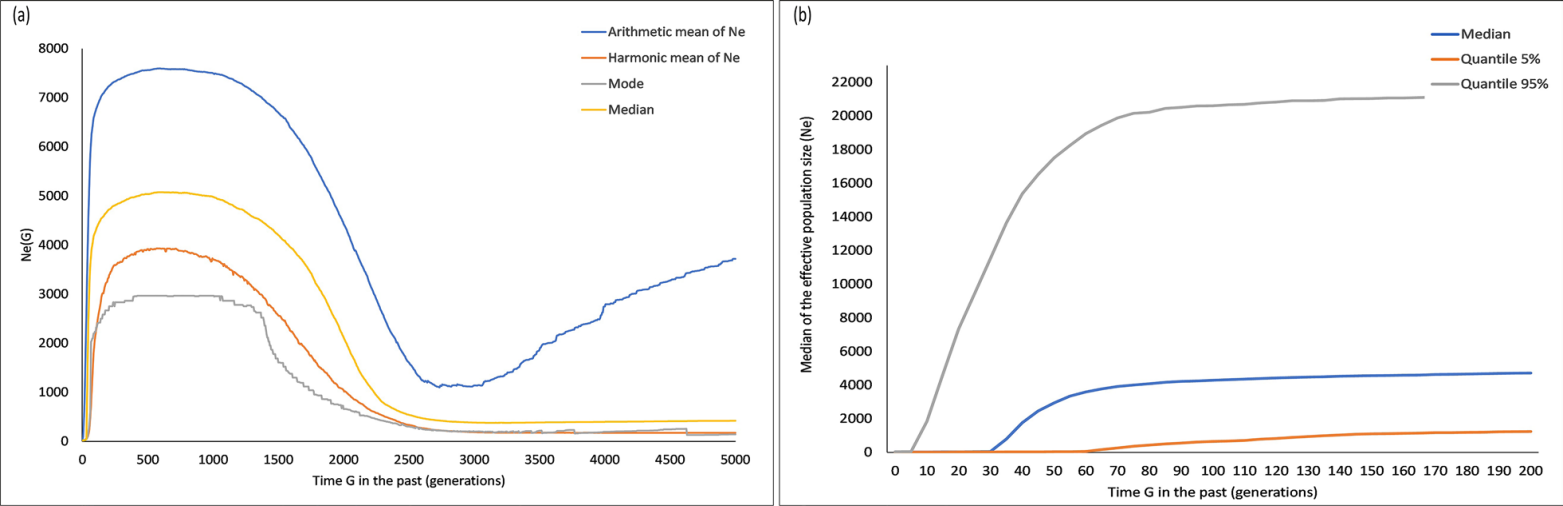
Estimates of effective population size (*Ne*) of *R. e. eldii* in KLNP based on 19 microsatellite loci using approximate likelihood MCMC approach in VarEff v.1.2 package in R (**a**) *Ne* estimates from present to 5000 generations in the past and (**b**) present to 200 generations in the past.

Estimates of past effective population size based on the approximate likelihood Markov chain Monte Carlo (MCMC) method obtained low *N*_*e*_ estimates until ~ 3000–5000 generations ago (median = 383–420) with a stable population trend until ~ 3000 generations (Fig. 5a). Subsequently, the population showed a massive increase in the effective population size for ~ 3000 generations until the recent bottleneck event was detected at ~ 300 generations ago (median = 4876) (Fig. 5a). The ancestral effective population size as inferred from the estimates of the posterior distribution revealed a large population size during the past ~ 300–1000 generations (median 4281–4978) which corresponds to around 1500–5000 Ybp, considering the generation time of *R. e. eldii* as 5 years^7^. The posterior distribution of time to the most recent common ancestor (T_MRCA_) supported the demographic estimates with the first peak observed at ~ 50 generations and a second peak at ~ 3000 generations, indicating the time at which the population underwent genetic bottlenecks (see Supplementary Fig. S6 online).

Assessment of the contemporary effective population size of wild population of *R. e. eldii* showed that the population underwent a steady decline during the past ~ 100–200 generations (median 4281–4717) (Fig. 5b) around 500–1000 Ybp. During the last ~ 30 − 100 generations, the decline accelerated and the population experienced a sudden, steep and continuous drop in the population size (median 45–4281), which accounts for a substantial decline in the effective population size of *R. e. eldii* during this period. However, the estimates of contemporary effective population size based on the linkage disequilibrium method were comparatively lower (median = 7.5, 95% CI 4.7–10.8) than those obtained using the MCMC approximation method (median = 20) (see Supplementary Table S6 online).

## Discussion

Demographic, environmental, and genetic factors profoundly affect the viability of populations^33^. Genetic diversity has potential impacts on the persistence and adaptation of populations to environmental changes^34^. Small populations generally exhibit low genetic variation due to population bottleneck and founder effects induced by natural as well as anthropogenic factors^1^. Eld's deer occur in small and fragmented populations, and its management largely depends on human-assisted maintenance of genetic diversity through population reintroductions. Hence, information on the genetic variability of Eld's deer populations across its distribution range is of prime importance for planning conservation strategies. This study examines the most extensive mtDNA control region dataset available to date, to provide information on the levels of genetic diversity, phylogeographic and phylogenetic patterns of Eld's deer subspecies.

Additionally, this is the first attempt to estimate the divergence events among the Eld's deer subspecies using complete mitochondrial genomes. Due to the unavailability of biological samples from other subspecies, we restricted the microsatellite analyses to *R. e. eldii* and delivered a comprehensive insight into the demographic history and past effective population size of this subspecies. The results of this study, combined with low population densities and increasing anthropogenic threats, raise serious conservation concerns and indicate increased susceptibility of Eld's deer to genetic, demographic, and stochastic factors.

### Phylogeography, divergence times and phylogenetics of *R. eldii*

The phylogeographic analyses based on the control region (447 bp) indicated the presence of genetic structure among the Eld's deer subspecies. Consistent with the wide geographical distribution, persistence in multiple populations, and comparatively stable demographic history, *R. e. thamin* exhibited the highest number of haplotypes (18) followed by *R. e. siamensis* (4) and *R. e. eldii* (1) which occurs in small and isolated populations (Fig. 2b). We observed no segregation of haplotypes based on sampling locations in *R. e. thamin and R. e. eldii*. However, in *R. e. siamensis* the haplotypes were unique to their respective analysed populations. Further, the captive and wild populations of *R. e. eldii* showed complete homogeneity in 447 bp of the control region. Contrary to previous phylogenetic studies^29,31^, our phylogenetic analysis did not support the monophyly of Eld's deer subspecies (Fig. 3). Instead, we observed a close phylogenetic association between *R. e. eldii* and *R. e. thamin,* which is consistent with the close geographical distribution of these two subspecies. Despite its phylogenetic affinity to *R. e. thamin*, *R. e. eldii* possesses splayed hooves, a unique morphological adaptation for marshy habitats^24^. The presence of unique control region haplotypes and the observed patterns of phylogeographic structuring (Fig. 2) indicate a substantial amount of genetic differentiation between the mainland and HI populations of *R. e. siamensis*. Phylogenetic reconstruction based on the control region also supported the reciprocal monophyly of HI and mainland populations of *R. e. siamensis* (Fig. 3). Additionally, the genetic differentiation measures (Fst) and nucleotide sequence variation indicated significant differentiation between these isolated populations of *R. e. siamensis* (see Supplementary Table S1 online).

Divergence analysis based on mitochondrial genomes also supported the differentiation patterns observed between the HI and mainland populations of *R. e. siamensis.* This subspecies exhibits two divergent mitochondrial lineages corresponding to the HI and mainland populations, which diverged during the Late Pleistocene ~ 0.2 Mya (95% HPD 0.1–0.2) (Fig. 4). The low sea levels during this period^35^ resulted in the emergence of land bridges, which would have facilitated the dispersal of *R. e. siamensis* from mainland China to Hainan Island. A rise in the eustatic sea levels during Holocene^35^ interrupted the terrestrial connectivity and would have resulted in isolating the Hainan Island from the mainland populations of *R. e. siamensis* (Fig. 4). During the periods of low sea levels of Plio-Pleistocene, the emergence of land bridges comprising of tropical forests and savannah corridors facilitated faunal dispersion between Indochina, Southeast Asia and the Sundaic islands^36^ possibly through the Siva-Malayan, Sino-Malayan and Taiwan-Philippine Archipelago corridors^37^. These events are supported by the patterns of genetic differentiation observed in this study between the isolated HI and mainland populations of *R. e. siamensis*. Therefore, intermixing the HI and mainland populations of *R. e. siamensis* to ameliorate the levels of genetic diversity is not a pragmatic proposal; instead, increasing the population size and establishment of meta-populations in the wild would practically benefit its long-term survival in the wild.

The divergence between *R. e. eldii* and *R. e. siamensis* took place around 0.4 Mya (95% HPD 0.3–0.5). During the Late Pleistocene and Early Holocene period, Northeast India was dominated by open grassland habitats^38, 39^, which would have facilitated the dispersal of grassland dependent species during the period. The dynamic influences of climatic and environmental factors during the Pleistocene have significantly driven the divergence and phylogeographic structure of several species^40^. We hypothesise that different ecological niches occupied by the Eld’s deer subspecies might have facilitated their diversification. Overall, the mitochondrial genomes investigated in this study revealed two major divergence events among the Eld's deer subspecies during Late Pleistocene. However, one limitation of our study is the unavailability of the complete mitochondrial genome of *R. e. thamin*, which would have provided more profound insights into the evolutionary history of Eld's deer.

Further, our results indicated diversification of two evolutionary lineages in tribe *Cerv*ini which occurred around 4.5 Mya (95% HPD 3.6–5.4) during Pliocene. The first lineage gave rise to species of the genus *Rucervus* and *Axis*, and the latter radiated into the genera *Rusa*, *Cervus*, *Dama,* and *Elaphurus* including the Eld’s deer (Fig. 4). Eld's deer is presently classified in the genus *Rucervus* based on the morphological similarities with *R. duvaucelii* and the now-extinct *R. schomburgki*^24^. However, these morphological similarities possibly characterise convergence rather than phylogenetic relatedness^41^. Moreover, our divergence and phylogenetic analyses revealed an ancestral divergence of *Axis* and *Rucervus* sp*.* from the main phylogenetic stock of tribe *Cervini* (Fig. 4 and Supplementary Fig. S3 online). Therefore, based on the significant evolutionary and systematic distance of Eld's deer from other species of the genus *Rucervus,* we propose to assign Eld's deer to genus *Cervus* as *Cervus eldii* together with *E. davidianus* and all four species of genus *Rusa* (*R. unicolor, R. timorensis, R. alfredi*, and *R. marianna*) (see Supplementary Fig. S3 online). These recommendations are in agreement with the previous molecular phylogenetic studies^26,27^. The classification proposed in this study will resolve the taxonomic perplexity of Eld's deer and other species of tribe *Cervini* to its phylogenetically most appropriate genus.

### Microsatellite genetic variation in *R. e. eldii*

The microsatellite analyses revealed low levels of genetic diversity in both captive and wild populations of *R. e. eldii* based on 19 microsatellite loci (see Supplementary Table S4 online). However, the wild population exhibits relatively higher levels of microsatellite genetic diversity compared to the captive populations, which is consistent with KLNP being the source population of all the captive stock. The population structure analysis using microsatellite revealed the presence of two genetic clusters (*K* = 2) and assigned the captive and wild populations to separate genetic clusters (see Supplementary Fig. S5 online). The captive population of *R. e. eldii* was established at Alipore Zoological Garden, West Bengal in 1956, by acquiring a pair of wild individuals from KLNP. All the existing populations in captivity are derived from the captive stock of Alipore Zoological Garden and have never been supplemented with individuals from the wild^42^. Hence, the assignment of all the captive individuals in a single cluster supports the evidence that all the captive populations are derived from the initial captive stock of Alipore Zoo. Further, no gene flow between the wild and captive populations and a small number of founder individuals of captive stock would have resulted in the observed patterns of genetic distinctiveness between the captive and wild populations of *R. e. eldii.*

The populations of *R. e. siamensis* in Datian, Bangxi, and Ganshiling Nature Reserves of Hainan Island were reported to exhibit low mean heterozygosity and genetic polymorphism estimates with *H*_*o*_ = 0.34 ± 0.03, 0.33 ± 0.01 and 0.36 ± 0.03 and *H*_*e*_ = 0.28 ± 0.01, 0.32 ± 0.00 and 0.36 ± 0.03, respectively^11^. The observed *H*_*o*_ and *H*_*e*_ for *R. e. eldii* in KLNP were estimated to be *H*_*o*_ = 0.31 ± 0.05 and *H*_*e*_ = 0.471 ± 0.033 (see Supplementary Table S4 online) which indicates that *R. e. eldii* in KLNP and *R. e. siamensis* in Hainan Island exhibit similar levels of microsatellite diversity. Both these subspecies have experienced severe range contraction during the last century and recovered from a small number of founder individuals^7^, therefore exhibit low levels of genetic polymorphism. Moreover, a recent study^43^ based on similar microsatellite markers estimated the mean heterozygosity (*H*_*o*_ = 0.42 ± 0.02 and *H*_*e*_ = 0.51 ± 0.03) of *A. porcinus*, which co-occurs with *R. e. eldii* in KLNP sympatrically. Both *A. porcinus* and *R. e. eldii* exhibit low to moderate levels of microsatellite genetic variability, which can be attributed to their single, small and isolated populations, inhabiting the space-constraint habitat in KLNP. Most of the selected microsatellite loci significantly deviated from the HWE, possibly due to the small population size, non-random mating, and impacts of inbreeding^44^ in *R. e. eldii*. In summary, the existing wild and captive populations of *R. e. eldii* exhibit low levels of microsatellite genetic diversity. The wild population in KLNP is highly susceptible to inbreeding because of the increased probability of interbreeding of related individuals due to its small and geographically isolated population.

### Past population changes in *R. e. eldii*

The low estimates of effective population size are congruent with the genetic bottleneck events detected in this study. The one-tailed Wilcoxon test for heterozygosity excess and allelic frequency distribution indicated that the wild population of *R. e. eldii* has gone through genetic bottleneck (see Supplementary Table S5 online). Posterior distribution of time to the most recent common ancestor (T_MRCA_) indicated two genetic bottleneck events (see Supplementary Fig. S6 online). The first event occurred ~ 3000 generations ago, which resulted in a mild fluctuation in the number of individuals (Fig. 5a). Subsequently, a massive upsurge in the effective population size was observed, indicating a range expansion event. Based on the generation time of Eld's deer (5 years)^7^, we assume that this event took place ~ 15,000 Ybp.

During the Late Pleistocene-Early Holocene, Northeast India had experienced dynamic fluctuations in climatic conditions, significantly affecting the past vegetation structure of the region^45–47^. The existing population of *R. e. eldii* inhabits the floating meadows of KLNP situated in the southern fringes of Loktak Lake in Manipur. The existing lake is the remnant of the original lake, which once occupied almost the entire Manipur valley^39^ and is known to have originated around 25,000 Ybp^48^. Since the course of its formation, the Loktak Lake underwent a prolonged phase of hydrodynamic and morphological changes^48^. Therefore, we presume that the lake during this period would have remained uninhabitable for *R. e. eldii*. The low estimates of effective population size during ~ 3000–5000 generations ago (median 383–420) around 15,000–25,000 Ybp are therefore consistent with the biogeographical history of the region. Subsequently, the appearance of open grasslands with aquatic herbaceous associates in the Loktak Lake region during Late Holocene^38,39^ indicates the emergence of extensive habitat conditions favourable for grassland dependent species, such as *R. e. eldii*. As shown in our results, an increase in the ancestral population size during the past ~ 2500–300 generations (median 540–4876) (Fig. 5a), signifies a range expansion event which would have been triggered due to the increased habitat availability during this period. Range expansion events and genetic drift often have negative impacts on the genetic diversity of the species^49,50^. Furthermore, our results indicated a second genetic bottleneck ~ 1500 Ybp (~ 300 generations ago; median = 4876)*,* which caused a steady decline in the population size till past ~ 100 generations (median = 4281) (Fig. 5b). Subsequently, the population underwent a rapid reduction in the effective population size during the past ~ 35–100 generations (median 791–4281). During this period, the Manipur valley underwent a drastic climatic transition, and warm and dry conditions replaced the prevailing warm and humid climatic conditions. Consequently, a reduction in the Loktak Lake dimensions along with the associated aquatic vegetation^38^ possibly reduced the habitat available for *R. e. eldii* which potentially resulted in a reduction in the effective population size. The intensification of human activities during the Late Holocene period is likely to accelerate the reduction and fragmentation of the suitable habitats of Eld's deer in the region. We hypothesise that the cumulative impacts of these factors would have influenced the demographic history of Eld's deer and consequently resulted in the reduction of genetic diversity.

### Implications for conservation and management

The findings of this study provide significant implications for planning conservation of Eld's deer across its distribution range. Our results demonstrate that *R. e. siamensis* exhibits two divergent mitochondrial lineage, with long divergence history and substantial amount of genetic differentiation. However, due to sampling restrictions, we were unable to either confirm or invalidate the subspecies status of Hainan's Eld's deer. We recommend a combined assessment of genetic variability between these populations to confirm the patterns of mtDNA variation highlighted in this study. If confirmed, the HI populations might represent a separate subspecies of Eld's deer. Until then, we recommend to identify the HI and mainland populations of *R. e. siamensis* as distinct units for conservation and suggest to prevent any intermixing of these lineages. As proposed by Zhang et al.^11^, we support the reintroduction and recovery programs for *R. e. siamensis* within the historical distribution range throughout the Hainan Island.

Low levels of genetic diversity, past effective population size, and widespread genetic bottleneck events in *R. e. eldii* accentuate its vulnerability to loss of evolutionary potential due to resulting genetic erosion, therefore necessitating immediate conservation interventions. The contemporary estimates of the effective population size of *R. e. eldii* are low (median = 7.5, 4.7–10.8 at 95% CI), far below the minimum threshold required to prevent inbreeding depression (*Ne* > 100) and to maintain long-term evolutionary potential (*Ne* > 1000) as proposed by Frankham et al.^51^. The HI populations of *R. e. siamensis* also contain low genetic diversity^11^, but large population size, which will undoubtedly benefit its population recovery in the wild. Unlike the HI populations, *R. e. eldii* exists as a single isolated wild population in a space-constrained habitat in KLNP with low population abundance in the wild^14^. Therefore, *R. e. eldii* is exceedingly susceptible to extinction as any further reduction in the number of breeding individuals will have detrimental effects on the viability of the population. Given these factors, it is crucial that improved conservation efforts with overarching strategies are implemented with immediate objectives to maintain population abundance in the wild as well as restoration of genetic diversity. Based on the observed patterns of genetic diversity and population genetic structure of *R. e. eldii*, we recommend initiating the conservation breeding using individuals from both captive and wild populations. Subsequently, the captive-bred founder stock can be used to ameliorate the levels of genetic diversity of the wild population by restocking the existing population in KLNP and establishing alternative populations to ensure its long-term persistence in the wild.

Furthermore, high human population density and increasing resource dependence, together with encroachment and changing land-use patterns in and around KLNP and Loktak Lake are leading to overexploitation of natural resources, thereby affecting the ecological integrity of the Park^14^. Therefore, planning immediate conservation strategies to mitigate the prevalent anthropogenic pressures, and arrest any further change in the existing land-use regime will benefit in protecting the ecological integrity of the Loktak Lake including KLNP, thereby ensuring the long-term survival of *R. e. eldii* in the wild. Any further delay in mitigating these conservation challenges may lead to an irreversible damage to the genetic diversity of the species.

The long-term survival of Eld's deer in the wild largely relies on human-assisted maintenance of genetic diversity through population reintroductions, restocking, and conservation breeding. However, the lack of range-wide information on genetic variation raises apprehensions on the effectiveness of these strategies. The need for mapping the spatial genetic variation using an extensive set of mitochondrial and nuclear markers cannot be overstated, especially given the critical status of Eld's deer across its distribution range. The information generated in this study will aid in evaluating the efficacy of the ongoing population recovery programs adopted for Eld's deer subspecies. Moreover, a collaborative approach among various institutions and policymakers across Eld's deer range countries is crucial to support the conservation efforts for ensuring the long-term survival of this species in the wild.

## Materials and methods

### Sampling and DNA extraction

We collected non-invasive biological samples of *R. e. eldii* including freshly defecated faecal pellets from Keibul Lamjao National Park (KLNP), Manipur (n = 48) and captive populations (n = 31) of Alipore Zoological Garden, West Bengal (n = 9), Manipur Zoological Garden, Manipur (n = 10), Assam State Zoo, Assam (n = 8) and National Zoological Park, New Delhi (n = 4) during 2006–2009 (see Supplementary Table S3 online). We also collected tissue samples (n = 3) from the remains of dead animals from KLNP during 2019 (see Supplementary Table S7 online) (Fig. 1). Tissue and faecal pellet samples were stored in absolute ethanol at − 20 °C for DNA extraction. Total genomic DNA from tissue and faecal samples were extracted using QIAamp DNA Blood and QIAamp DNA Stool kit (QIAGEN) respectively following the manufacturer's protocols. Tissue samples were used to sequence the complete mitochondrial genomes and faecal pellets for sequencing the mtDNA control region and microsatellite analyses of *R. e. eldii*. The tissue samples were obtained from remains of naturally dead animals and therefore, did not require the approval of the animal ethical committee. The samples were collected with due permissions from the Principal Chief Conservator of Forests and the Park administration of Manipur. All the field and laboratory experiments were executed under the pertinent guidelines.

### PCR and sequencing

We sequenced the control region (447 bp) using *Cerv.tPro*: 5′-CCACYATCAACACCCAAAGC-3′ and *CervCRH*: 5′-GCCCTGAARAAAGAACCAGATG-3′^29^. Sequencing of the mitochondrial genome was performed using 23 pairs of primers (see Supplementary Table S8 online) described by Hassanin et al.^52^ with an overlapping region of 80–120 base pairs to remove any gaps among the amplicons. Due to the hyper-variable nature of the control region, bi-directional sequencing using both forward and reverse primers was performed. Polymerase Chain Reactions (PCR) for both control region and mitogenome amplifications were carried out in 20 µL volumes containing 10–20 ng of the total genomic DNA, 10 µL of 2× PCR buffer (Applied Biosystems), 0.2 mg/mL Bovine Serum Albumin (BSA), 0.3 µM dNTPs, 0.3 µM of forward and reverse primers with 0.1 µL (0.5 units) of DreamTaq DNA Polymerase (ThermoFisher Scientific). PCR conditions followed an initial denaturation at 94 °C for 5 min, followed by 35 cycles at 95 °C for 35 s, 55 °C for 45 s, and 72 °C for 1 min 30 s. Reactions were terminated with a final extension of 10 min at 72 °C. We used positive and negative controls to monitor the efficacy and reliability of the reactions. PCR products were visualised for positive amplicons using 2% agarose gel electrophoresis using ethidium bromide and were examined under UV illuminator. Positive amplicons of control region and 23 fragments of the mitochondrial genomes were sequenced using Sanger sequencing in ABI 3500XL Genetic Analyser using BigDye v.3.1 sequencing kit (Applied Biosystems).

### Microsatellite amplification and genotyping

We used 19 microsatellite loci^53–63^ (see Supplementary Table S4 online) for assessing the genetic variability in the wild (n = 48) and captive populations of *R. e. eldii* (n = 31) using non-invasively collected fresh faecal pellet samples. PCR was performed in 10 µL reaction volumes with 2.0–3.0 µL of total genomic DNA, 5 µL of QIAGEN Multiplex PCR Buffer Mix (QIAGEN) and 0.2 µM of each labelled forward and 0.2 µM of the non-labelled reverse primers. PCR cycles of the microsatellite loci were performed at an initial denaturation at 94 °C for 15 min followed by 40 cycles of 94 °C for 30 s with annealing temperatures ranging from 48 to 60 °C for 45 s, 72 °C for 40 s, and final elongation at 72 °C for 30 min. The products were then genotyped in ABI 3130 Genetic Analyser using LIZ 500 Size Standard (Applied Biosystems). Positive and negative controls were used to monitor the efficacy and reliability of the reactions.

## Data analyses

### Phylogeography and genetic differentiation

We obtained a global alignment of 200 control region sequences (447 bp) representing all the three subspecies of Eld's deer (see Supplementary Table S3 online). The dataset was comprised of published control region sequences downloaded from GenBank (n = 150) along with 24 unique individuals identified in the wild population of *R. e. eldii* out of total 48 faecal pellet samples collected from KLNP and captive populations (n = 23) from Alipore Zoological Garden (n = 9) Manipur Zoological Garden (n = 10) and National Zoological Park (n = 4) using 19 microsatellite markers. We also included the control region sequences (n = 3) obtained from the complete mitochondrial genomes sequenced in this study. The faecal pellet samples obtained from Assam State Zoo (n = 8) were excluded from phylogeographic analysis because they failed to amplify at the control region. The control region sequences were aligned using the CLUSTAL W algorithm^64^ in program MEGA X^65^. Haplotype (*hd*) and nucleotide diversities (*Pi*) were calculated using program DnaSP v.5.10^66^. Haplotypes were derived by removing non-informative sites, and gaps were considered. The spatial relationship among the haplotypes was reconstructed using the median-joining network in PopART v.1.7^67^. Bayesian Analysis of Population Structure (BAPS) v.6.0^68,69^ was used to identify the number of genetic clusters. We performed the non-spatial clustering with admixture analysis using an assumed number of clusters (*K*) ranging from 1 to 30 with ten replicates for each value of *K* and 500 iterations. Pairwise genetic differentiation (Fst) of the identified clusters were calculated using program DnaSP v.5.10^66^. Phylogenetic relationships among the Eld's deer subspecies were reconstructed using the partial fragment of the control region based on the Bayesian approach as implemented in program MrBayes v.3.2^70^. The most appropriate model for nucleotide substitution was selected based on the Akaike Information Criterion (AIC)^71^ values using program jModelTest v.2.1.3^72,73^. We performed two MCMC chains of 30 million simulations sampling at every 10,000 generations and discarded the initial 3 million runs as burn-in. The output tree topologies were edited using FigTree v.1.4.2 (http://tree.bio.ed.ac.uk/sofware/fgtree/).

### Divergence times and phylogenetic analyses

The complete mitochondrial genome of 16, 357 bp sequenced from three individuals of *R. e. eldii* were obtained by aligning the 23 overlapping sequence fragments using SeqScape software v.3.0 (Applied Biosystems). The alignments were visualised manually and Phred Quality Score of each nucleotide variation (Q ≥ 20) was selected as a threshold for considering the variable sites (https://apps.thermofisher.com/apps/spa/#/apps). The mitochondrial genome annotation was performed in CGView Server^74^. The complete mitochondrial sequences were submitted to GenBank (Accession No: MT555112–MT555114). Further, we included the published complete mitogenomes of 10 species of tribe *Cervini*, *Bos javanicus,* and *Muntiacus reevesi* obtained from GenBank to estimate the divergence events and reconstruct the accurate phylogenetic position of Eld's deer (see Supplementary Table S7 online). We used 13 PCGs, two rRNA genes and control region sequences to perform the divergence and phylogenetic analyses. All the analysed mitochondrial genes were aligned separately using CLUSTAL W algorithm^64^ in program MEGA X^65^. Gaps were considered in the sequence alignments of each gene because of the variable lengths of PCGs and control region sequences among the analysed species. The aligned sequences of the mitochondrial genes were concatenated in MEGA X^65^ to obtain the final alignment for performing further data analyses. The degree of substitution saturation was calculated using program DAMBE to test whether the observed entropy is significantly lower than the entropy of full substitution saturation in the analysed sequences^32,75^. Divergence times were estimated using the Bayesian approach as implemented in program BEAST v.1.8.2^76^. PartitionFinder^77^ was used for selecting the most appropriate data partitioning scheme of nucleotide substitution for evolutionary rate heterogeneity. Greedy algorithm with linked branch length along with Bayesian Information Criterion as implemented in PartitionFinder was used. Substitution models were selected with separate model estimation for 13 PCGs using different codon positions and individually for rRNA and control region sequences. We used fossil information of the oldest known fossils of (1) Cervidae (18.4 Mya)^78^ for the Bovidae/Cervidae split and tribe *Muntiacini* (8 Mya)^79^ as calibration points. We used the lognormal uncorrelated relaxed-clock model and Yule speciation process with normally distributed prior parameters. We performed two independent divergence analyses with 50 million generations, sampling at every 1000 generations with a 10% burn-ins.

Phylogenetic analysis to reconstruct the molecular phylogeny of tribe *Cervini* based on 13 PCGs, two rRNA and control region sequences was executed in MrBayes v.3.2^70^. The best suitable model for nucleotide substitution was obtained based on the lowest AIC values using jModelTest v.2.1.3^72, 73^. We ran two MCMC chains of 50 million simulations sampling every 10,000 generations. We discarded the initial 5 million runs as burn-ins. Parameter convergence and effective sample size (> 400) for divergence and phylogenetic analyses were analysed in program TRACER v.1.6. TREEANNOTATOR v.1.8.2 (BEAST package) was used for annotating the output tree with a burn-in of 10%. The output tree topologies were visualised and edited in FigTree v.1.4.2 (http://tree.bio.ed.ac.uk/sofware/fgtree/).

### Microsatellite genetic variation

We performed the identification of alleles using GeneMarker v.2.7.4^80^. The likelihood of two unrelated individuals and siblings sharing the same genotype (probability of identity, *P*_*ID*_ and probability of identity for siblings, *P*_*ID-Sibs*_) was executed in Gimlet v.1.3.3^81^. The overall summary statistics comprising of the total number of alleles per locus (*Na*), observed (*H*_*o*_) and expected (*H*_*e*_) heterozygosity, and deviations from the Hardy–Weinberg equilibrium (HWE) were calculated in GenAlEx v.6.0^82^. The Polymorphic Information Content (PIC) values were estimated in program CERVUS v.3.0.7^83^. We used model-based hierarchical Bayesian clustering approach implemented in program STRUCTURE v.2.3.4^84^ to identify the presence of genetic structure in *R. e. eldii* populations. We used an assumed number of clusters (*K*) ranging from 1 to 10. For each *K*, we executed ten iterations with a burn-in of 50,000 followed by 5,00,000 MCMC simulations under the admixture model with correlated allele frequencies. The optimal number of clusters in the sampled populations were determined following Evanno et al.^85^ approach executed in the web version of STRUCTURE HARVESTER v.0.6.94^86^. The assignment probability plot using individual membership coefficient was produced using program DISTRUCT v.1.1^87^. Further, the genetic differentiation estimates Fst, Gst and Jost’s D among the identified clusters were estimated using program *strataG*^88^ package in R with 1000 bootstrap iterations.

### Past effective population size

To detect the signatures of bottleneck events, we performed the transient heterozygosity excess and mode-shift tests in program BOTTLENECK v.1.2.02^89^ assuming that microsatellite mutations followed the Infinite Allele Model (IAM), Stepwise Mutation Model (SMM) or Two-phase Model (TPM). In TPM we used 95% single-step mutational events at 12% variance^89^. We also performed the one-tailed Wilcoxon test to determine the significance of the heterozygosity excess. The allelic distribution curve was analysed to determine whether the population is under mutation-drift equilibrium (L shaped curve) or not (mode shift)^90, 91^.

We implemented the linkage disequilibrium method^92–94^ to estimate the contemporary effective population size using the bias-corrected version in program NeEstimator v.2^95^. The linkage disequilibrium approach is the most suited method for microsatellite data which has been proven to be relatively robust in assessing the effective population size^94^. We also implemented the approximate likelihood MCMC approach using VarEff v.1.2 package in R (https://qgsp.jouy.inra.fr/)^96^ to estimate the past changes in effective population size. This approach implements coalescence theory to generate posterior distributions of effective population size using an approximation of likelihood based on different *prior* demographic parameters. It implements the distribution of motif distance frequencies between alleles at the tested microsatellite loci^96, 97^. This method allows recovering the posterior distribution of the time to the most recent common ancestor (T_MRCA_) between two alleles and provides the time of the bottleneck events in the number of generations in the past. We implemented a Two-phase Mutation Model (TPM) to estimate the past changes in the population size with proportion for multi-step mutations of c = 0.2 and an average microsatellite mutation rate (μ) of 2.0 × 10^–3^ per generation^98–103^. We estimated the effective population size from present to 5000 generations in the past, assuming the generation time (G) of 5 years for Eld's deer^7^. Priors for past effective population size were derived from global estimates of Theta (θ = 4Neμ) executing the Theta function in VarEff^96^. MCMC runs were performed using 10,000 batches with sampling at every ten batches and a burn-in of 10,000. We performed trial runs by changing the analysis parameters and finally selected the parameters recommended by Nikolic and Chevalet^96^ (see Supplementary Table S9).

## Supplementary Information

Supplementary Information 1.

Supplementary Information 2.

## Data Availability

The sequence data generated in this study is submitted to the GenBank database, National Centre for Biotechnology Information under Accession Nos. MT555259–MT555301, MW033296–MW033299 and MT555112–MT555114. The genotyping data used in this study is available in the supplementary section.
