# Supplementary material for: Population genetics and evolutionary history of the endangered Eld’s deer (Rucervus eldii) with implications for planning species recovery
Source: Sci Rep. 2021 Jan 28;11:2564. doi: 10.1038/s41598-021-82183-7 (PMC7844053; doi:10.1038/s41598-021-82183-7)
Supplement: Supplementary file 1 — Supplementary Information 1. [file 41598_2021_82183_MOESM1_ESM.pdf]

Population genetics and evolutionary history of the endangered Eld's deer (*Rucervus eldii*) with implications for planning species recovery

Mirza Ghazanfarullah Ghazi[1], Surya Prasad Sharma[1], Chongpi Tuboi[1], Sangeeta Angom[1], Tennison Gurumayum[1], Parag Nigam[1], Syed Ainul Hussain[1*]

[1] Wildlife Institute of India, Post Box #18, Chandrabani, Dehra Dun, 248002, Uttarakhand, India.

[*]**Corresponding author-** Syed Ainul Hussain, E-mail- hussain@wii.gov.in

Table S1. Pairwise F_{ST} and sequence variation values among the Eld's deer subspecies**.** F_{ST} values are mentioned below the diagonal and nucleotide sequence variation values above the diagonal (P<0.05).

	R. e. eldii	*R. e. siamensis* (Hainan)	*R. e. siamensis* (Mainland)	*R. e. thamin*
R. e. eldii	-	0.035	0.040	0.028
R. e. siamensis (Hainan)	1.00	-	0.027	0.027
R. e. siamensis (Mainland)	0.539	0.453	-	0.039
R. e. thamin	0.523	0.448	0.347	-

Table S2. Divergence dates with median node ages and 95% Highest Posterior Densities (HPD).

**Divergence events**	**Node age (in million years [Mya])**			**Other studies**
	**Median**	**Minimum**	**Maximum**	
Bovidae and Cervidae	18.01	15.1	20.8	18.4 [78]
Cervini and *Muntiacini*	7.81	6.3	9.2	7.9 [26]
Axis-Rucervus and *Rusa-Cervus-Dama-Elaphurus*	4.54	3.6	5.4	5 [26]
Cervus-Rusa and *R. eldii- Elaphurus*	2.73	2.1	3.3	2.9 [26]
R. eldii and *Elaphurus*	2.01	1.5	2.5	2.1 [26]
R. e. eldii and *R. e. siamensis*	0.44	0.3	0.5	
R. e. siamensis (Mainland and Hainan Island populations)	0.2	0.1	0.2	

Table S3. Details of the samples and GenBank accession numbers of control region sequences used in this study.

Subspecies	Location	Samples (*n*)	References	GenBank Accession No.
R. eldii siamensis	Hainan Island, China	55	Pang et al., 2003 [104]	AF359286-AF359340
	Hainan Island, China	2	Balakrishnan et al., 2003 [29]	AY137115-AY137116
	Hainan Island, China	11	Zhang-2009 [12]	FJ851215-FJ851225
	Hainan Island, China	1	Kong and Li, 2010, Unpublished	HM138200
	Hainan Island, China	1	Kong and Li, 2010, Unpublished	NC014701
	Dusit Zoo, Thailand	4	Balakrishnan 2003 [29]	AY137080-AY137083
	Dusit Zoo, Thailand	2	Zhang-2009 [12]	FJ851238-FJ851239
	Paris Zoo, France	2	Randi et al., 2001 [105]	AF291892-AF291893
		1	Hassanin et al., 2012 [27]	JN632697
R. eldii thamin	Thailand	12	Zhang-2009 [12]	FJ851226-FJ851237
	Thailand	9	Wajjwalku et al., 2009, Unpublished	GQ292465-GQ292473
	Thailand	15	Wajjwalku et al., 2014, Unpublished	KM881597-KM881611
	Khao Kheo Open Zoo, Thailand	2	Balakrishnan et al., 2003 [29]	AY137084-AY137086
	Conservation & Research Centre, USA	9	Balakrishnan et al., 2003 [29]	AY137085, AY137087-AY137090, AY137122-AY137125
	Yangon Zoo, Myanmar	12	Balakrishnan et al., 2003 [29]	AY137103-AY137114
	Chatthin Wildlife Sanctuary, Myanmar	12	Balakrishnan et al., 2003 [29]	AY137091-AY137102
R. eldii eldii	National Zoological Park, New Delhi	4*	This study	MW033296-MW033299
	Alipore Zoological Garden, West Bengal	9	This study	MT555259-MT555267
	Manipur Zoological Garden, Manipur	10*	This study	MT555268-MT555277
	Assam State Zoo, Assam	8*	This study	-
	Keibul Lamjao National Park, Manipur	48*	This study	MT555278-MT555301

*Samples from these populations were used for microsatellite genetic variation and estimation of past effective population size analyses.

Table S4. Measures of genetic variability of wild and captive populations of *R. e. eldii* based on 19 microsatellite loci. (*N*=No. of samples, *Na*=No. of observed alleles, H_o=Observed heterozygosity, H_e=Expected heterozygosity, F=Fixation Index, *HWE*= Hardy–Weinberg Equilibrium, P_{ID}=Probability of identity and $P_{ID(Sibs)}$=Probability of identity for siblings). Key: ns=not significant, SE=Standard error. * P<0.05, ** P<0.01, *** P<0.001.

**Location**	**Wild population (KLNP)**								**Captive populations**					
Locus	*N*	*Na*	H_o	H_e	*F*	*HWE*	P_{ID} *(biased)*	P_{ID} *(sibs)*	*N*	*Na*	H_o	H_e	*F*	*HWE*
AY302223 [54]	24	3	1.00	0.52	-0.92	***	3.75 × 10-1	5.94× 10-1	18	4	0.50	0.67	0.26	***
BM4208 [55]	24	3	0.46	0.60	0.24	***	7.22 × 10-2	2.83× 10-1	18	2	0.11	0.35	0.68	**
RT6 [63]	24	3	0.50	0.43	-0.15	***	7.34× 10-3	1.12× 10-1	18	2	0.28	0.24	-0.16	ns
INRA011 [59]	24	2	0.13	0.19	0.33	ns	1.87× 10-3	5.81× 10-2	18	4	0.39	0.49	0.20	***
RT1 [63]	24	3	0.17	0.45	0.63	***	4.30× 10-4	2.93× 10-2	18	1	0.00	0.00	-	
Ca42 [54]	24	3	0.33	0.54	0.38	***	4.22× 10-5	1.16× 10-2	18	2	0.00	0.44	1.00	***
Cervid [57]	24	2	0.38	0.50	0.25	ns	5.46× 10-6	4.89× 10-3	18	2	0.33	0.44	0.25	ns
RT27 [63]	24	3	0.04	0.51	0.92	***	6.59× 10-7	2.03× 10-3	18	2	0.17	0.31	0.47	*
BM6506 [55]	24	2	0.04	0.48	0.91	***	6.87× 10-8	8.08× 10-4	18	2	0.11	0.48	0.77	**
OarFCB193	24	3	0.04	0.59	0.93	***	6.30× 10-9	3.14× 10-4	18	2	0.00	0.28	1.00	***
T156 [58]	24	3	0.42	0.57	0.27	***	6.80× 10-11	5.01× 10-5	18	2	0.17	0.24	0.30	ns
CelJP27 [56]	24	4	0.50	0.66	0.24	***	7.02× 10-12	1.99× 10-5	18	4	0.06	0.51	0.89	***
DF/R [58]	24	4	0.54	0.74	0.27	***	5.29× 10-13	7.44× 10-6	17	1	0.00	0.00	-	
T193 [58]	24	3	0.29	0.59	0.50	***	4.73× 10-14	2.87× 10-6	18	2	0.28	0.24	-0.16	ns
BM4107 [55]	23	2	0.09	0.29	0.70	***	1.62× 10-16	2.52× 10-7	18	1	0.00	0.00	-	
MAF70 [61]	24	2	0.21	0.30	0.32	ns	3.84× 10-17	1.28× 10-7	18	2	0.00	0.49	1.00	***
L23481 [60]	24	2	0.33	0.33	-0.01	ns	4.96× 10-18	5.40× 10-8	18	2	0.00	0.49	1.00	***
AF232760 [53]	24	2	0.29	0.30	0.04	ns	1.11× 10-18	2.69× 10-8	18	1	0.00	0.00	-	
T507 [58]	24	2	0.13	0.35	0.65	**	6.96× 10-16	5.02× 10-7	18	2	0.00	0.20	1.00	***
Mean		2.68	0.31	0.47	0.34					2.11	0.13	0.31	0.57	
SE		0.15	0.05	0.03	0.10					0.21	0.04	0.05	0.09	

Table S5. Bottleneck detection in *R. e. eldii* population of Keibul Lamjao National Park, Manipur demonstrating (He < Heq) mutation–drift equilibrium obtained using IAM, TPM and SMM mutation models. *He*=Expected heterozygosity; *Heq*= Heterozygosity equilibrium; *Prob*=Probability; IAM=Infinite allele model; TPM=Two-phase model; SMM=Stepwise mutation model.

	**Observed**	**IAM**		**TPM**		**SMM**	
Loci	***He***	***Heq***	***Prob***	***Heq***	***Prob***	***Heq***	***Prob***
AY302223	0.531	0.385	0.234	0.478	0.436	0.478	0.426
BM4208	0.614	0.374	0.07	0.472	0.151	0.48	0.174
RT6	0.443	0.364	0.392	0.473	0.346	0.487	0.313
INRA011	0.191	0.213	0.488	0.255	0.445	0.263	0.435
RT1	0.465	0.362	0.378	0.477	0.362	0.483	0.351
Ca42	0.55	0.377	0.178	0.476	0.341	0.48	0.362
Cervid	0.51	0.218	0.029	0.26	0.027	0.263	0.042
RT27	0.52	0.371	0.258	0.473	0.458	0.489	0.51
BM6506	0.488	0.219	0.097	0.257	0.144	0.263	0.138
OarFCB193	0.598	0.366	0.094	0.478	0.19	0.482	0.189
T156	0.585	0.367	0.093	0.465	0.205	0.481	0.234
CelJP27	0.676	0.475	0.106	0.595	0.262	0.611	0.317
DF/R	0.756	0.482	0.004	0.599	0.004	0.608	0.009
T193	0.6	0.377	0.091	0.477	0.19	0.48	0.184
T507	0.361	0.22	0.277	0.264	0.384	0.264	0.37
BM4107	0.294	0.218	0.342	0.258	0.448	0.268	0.47
MAF70	0.311	0.217	0.317	0.257	0.422	0.267	0.44
L23481	0.337	0.213	0.28	0.256	0.386	0.268	0.412
AF232760	0.311	0.228	0.359	0.251	0.407	0.262	0.438
One-tailed Wilcoxon Test (*P*<0.001)		0.00000		0.00001		0.00004	

Table S6. Estimates of the present (N_{pre}) and past (N_{past}) (1000 generations ago) effective population sizes of wild population of *R. e. eldii* in Keibul Lamjao National Park, Manipur derived using VarEff.

**Effective Population Size**	**NeEstimator**	**VarEff**	
	N_{pre}	N_{pre}	N_{past}
Arithmetic Mean	-	29	7498
Harmonic Mean	-	14	3701
Mode	-	20	2965
Median	7.5	20	4978
5% quantile	4.7	6	1338
95% quantile	10.8	39	21962

Table S7. Details of sequences, references and GenBank accession numbers of complete mitochondrial genome sequences used in this study.

Species/subspecies	Reference	GenBank Accession No.
Rucervus duvaucelii (Swamp deer)	Hassanin et al., 2012 [27]	JN632696
Axis axis (Chital)	Hassanin et al., 2012 [27]	NC020680
Axis porcinus (Hog deer)	Hassanin et al., 2012 [27]	JN632600
Dama dama (European fallow deer)	Hassanin et al., 2012 [27]	NC020700
Dama mesopotamica (Persian fallow deer)	Hassanin et al., 2012 [27]	NC024819
Rusa alfredi (Philippine spotted deer)	Hassanin et al., 2012 [27]	JN632698
Rusa unicolor (Sambar deer)	Liu et al., 2018 [106]	KY946815
Rusa timorensis (Timor deer)	Hassanin et al., 2012 [27]	JN632699
Cervus elaphus (Red deer)	Wada et al., 2006 (unpublished)	NC007704
Cervus albirostris (White-lipped deer)	Hassanin et al., 2012 [27]	JN632690
Cervus nippon (Sika deer)	Wada et al., 2006 (unpublished)	NC006973
Elaphurus davidianus (Père David's deer)	Kong et al., 2011 (unpublished)	JN399997
Rucervus eldii eldii (Manipur's brow-antlered deer)	This study	MT555112*
	This study	MT555113*
	This study	MT555114*
Rucervus eldii siamensis (Siamese brow-antlered deer)	Hassanin et al., 2012 [27]	JN632697
Rucervus eldii siamensis (Hainan Eld's deer)	Kong et al., 2010 (unpublished)	HM138200
Rucervus eldii siamensis (Hainan Eld's deer)	Kong et al., 2010 (unpublished)	NC014701
Muntiacus reevesi (Chinese muntjac)	Zhang et al., 2002 (unpublished)	AF527537
Bos javanicus (Banteng)	Hassanin et al., 2012 [27]	JN632606

*Partial fragment of control region of these sequences was used in control region analysis.

Table S8. Details of twenty-three pairs of primers with overlapping fragments [52] used to sequence the complete mitochondrial genome of *R. e. eldii*.

Sr. No	Primer Id	Primer Sequence (5' → 3')	Forward/Reverse
1	DLU405	ACCATGCCGCGTGAAACCAGCA	F
2	12SL41	GYGYGGATRCTTGCATGTGTA	R
3	U1230	CACTGAAAATGCCTAGATGAG	F
4	L2226	CTAGGTGTAAACTAGRTGCTT	R
5	12SU829	GCACGCACACACCGCCCGTCAC	F
6	16SL518	CGCTTTCTTAATTGRTGGCTGC	R
7	16SU365	AGCCTGGTGATAGCTGGTTGTCC	F
8	16SL1056	AAGCTCCATAGGGTCTTCTCGTC	R
9	16SU946	CCGTGCAAAGGTAGCATAATCA	F
10	N1L64	CCTAGNACTTTTCGTTCNACTA	R
11	Uleu	GTGGCAGAGCCCGGTAATTG	F
12	IleL	TTACTCTATCAAAGTAACTC	R
13	N1U840	TYCGAGCATCHTAYCCHCGATT	F
14	N2L492	TGGTTTAGBCCBCCTCAKCCYCC	R
15	N2U354	CACTTYTGAGTNCCAGAAGT	F
16	AsnL	TAGGGTRTTTAGCTGTTAAC	R
17	TrpU	AGACCAAGAGCCTTCAAAGC	F
18	C1L339	GCTTCWACTATDGADGATGC	R
19	C1U246	GGNGGNTTYGGHAAYTGACT	F
20	C1L1017	GAARATRAAGCCTAGRGCTCA	R
21	C1U897	TTYACHGTHGGAATAGAYGT	F
22	C2L15	GCRTCTTGRAANCCTARTTG	R
23	SerU	CCCCCYAYWRYTGGTTTCAAGCCA	F
24	A8L1	GTKGAYGTRTCTAGTTGYGGCAT	R
25	C2U603	CAATGCTCHGARATYTGYGG	F
26	C3L45	GANARDGCTCCYGTDAGNGGTCA	R
27	A6U654	GCCTAYGTNTTYACYCTNCTAGT	F
28	GlyL	TGATTGGAAGTCARYTGTAC	R
29	C3U780	GTHTCYATCTATTGATGAGG	F
30	N4L27	CAGGTYAGRGGDATDAGTAT	R
31	U213M1	AGCYTGYGAAGCAGCACTAGG	F
32	L918M1	GCKGTRGCTCCTATRTARCTTCA	R
33	N4U840	AGCTCHATYTGYYTHCGYCAAAC	F
34	Leu2L	CCAATTTTTTGGYTCCTAAGRCC	R
35	Ser2U	CCGAAAAAGYAYGCAAGAACTGC	F
36	N5L652	GCDGATTTTCCDGTKGCDGCTA	R
37	N5U501	GACGARCAGAYGCHAAYACAGC	F
38	N5L1214	GTDAKTADDAGGGCTCAGGCG	R
39	N5U1146	GGMAGCCTNGCNYTAACAGG	F
40	N6RL154	AGTTTAATGGDHTDGGDGATTG	R

Table S8 continued. Details of twenty-three pairs of primers with overlapping fragments [52] used to sequence the complete mitochondrial genome of *R. e. eldii*.

Sr. No	Primer Id	Primer Sequence (5’ → 3’)	Forward/Reverse
41	N6RU102	CCATAACTRTAYAAAGCHGCAA	F
42	CBL402	CCTCARAATGATATTTGKCCTCA	R
43	CBU162	CAGGMCTATTCCTRGCHATACA	F
44	LTHR	CCCTTYTCTGGTTTACAAGACC	R
45	U1068	CATCGGACAACTAGCATCTAT	F
46	L482	CCTGAAGWAAGAACCAGATG	R

Table S9. Detail of the parameters used to estimate the past effective population sizes (Ne) of wild population of *R. e. eldii* in VarEff analysis (Nikolic and Chevalet, 2014). NBLOC=number of loci, JMAX=number of times that Ne changed in the past, MODEL=two phase mutation model (TPM), MUTAT=mutation rate, NBAR=prior value for Ne, VARP1=variance of the prior Ne, RHOCORN=coefficient of correlation between effective sizes in successive intervals, GBAR=number of generations since population origin, VARP2=variance of the prior of number of generations, DMAX=maximum distance (in motifs) between alleles, Diagonale=smoothing parameter, AccRate=acceptance rate (Nikolic and Chevalet, 2014) [96].

Population	NBLOC	JMAX	MODEL	MUTAT	NBAR	VARP1	RHOCORN	GBAR	VARP2	DMAX	Diagonale	AccRate
R. e. eldii (KLNP)	19	4	TPM	0.002	1500	3	0	5000	3	14	0.5	0.25

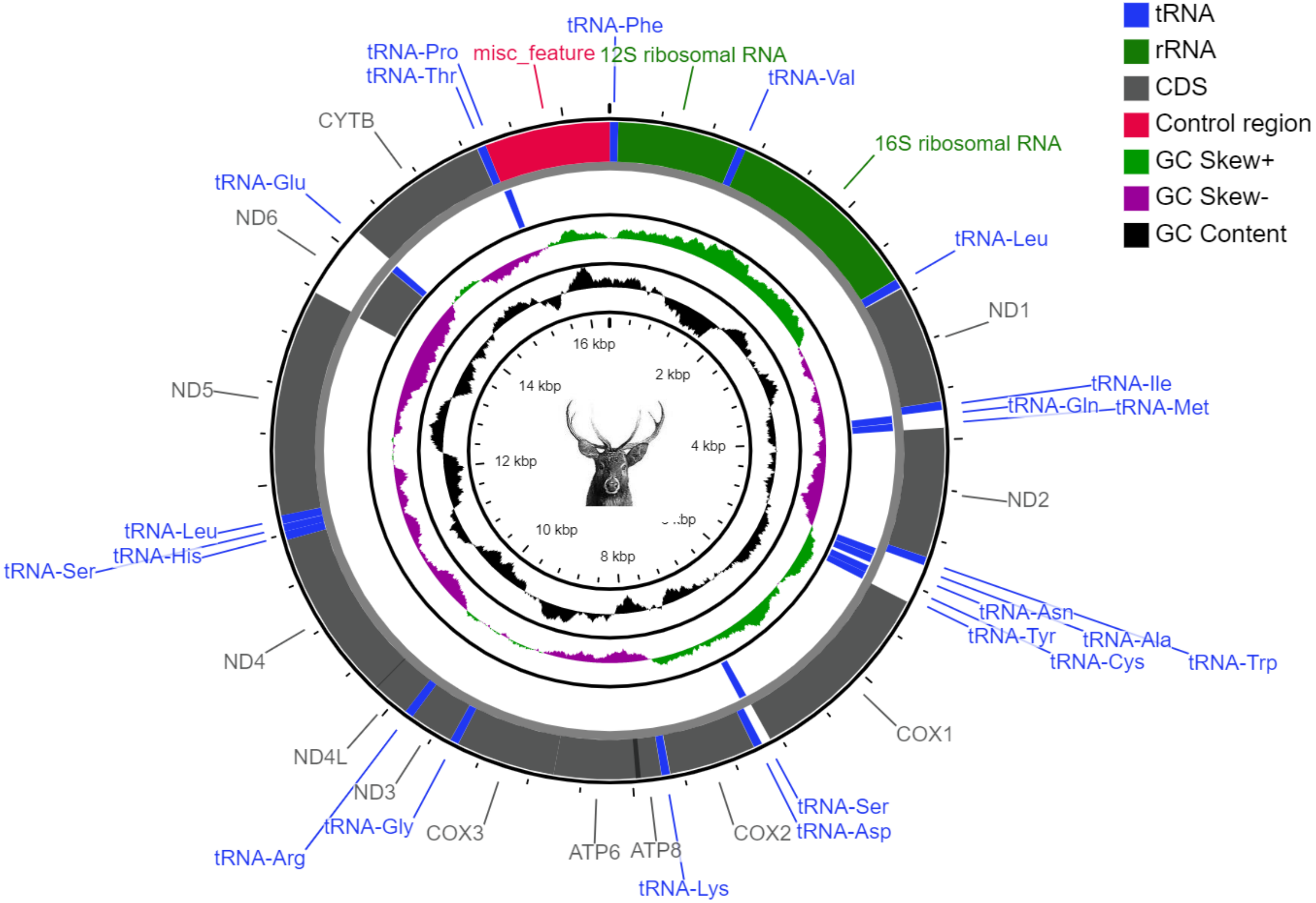

Figure S1. Graphical representation of the gene order and arrangement of complete mitochondrial genome of *R. e. eldii* sequenced in this study.

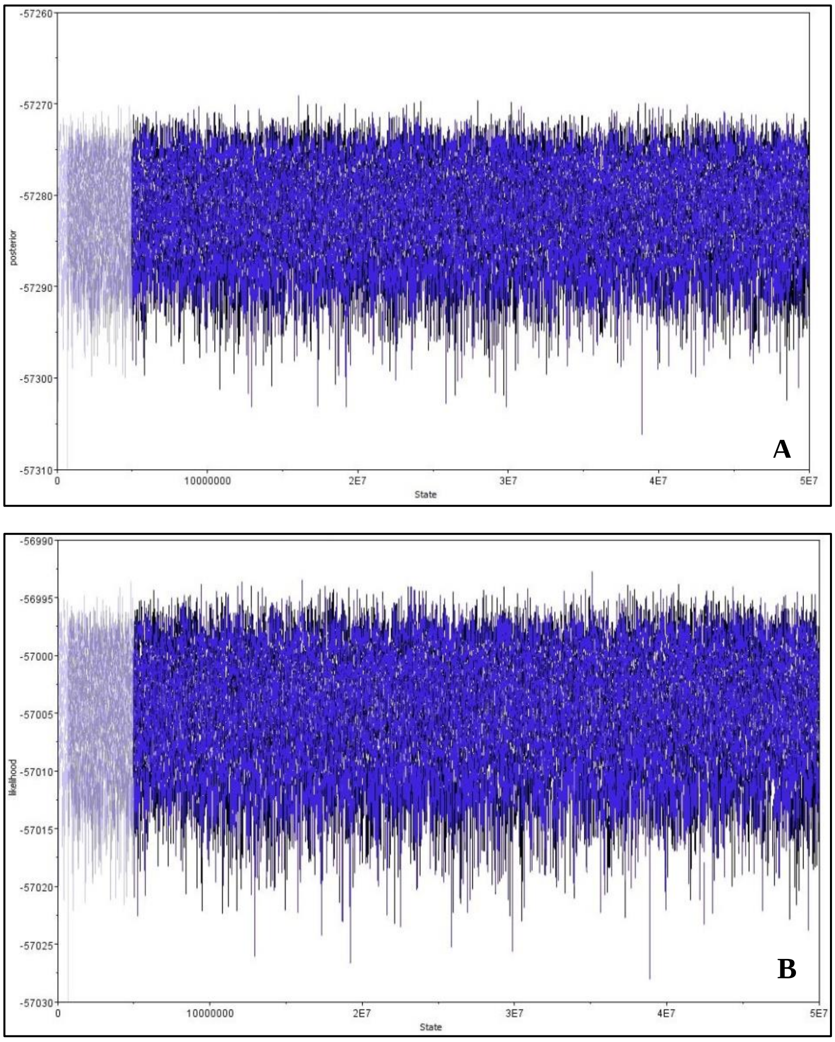

Figure S2. Detail of parameter convergence of (A) Posterior (ESS=8398), (B) Likelihood (ESS=10031) distribution of the two independent divergence analysis obtained using TRACER.

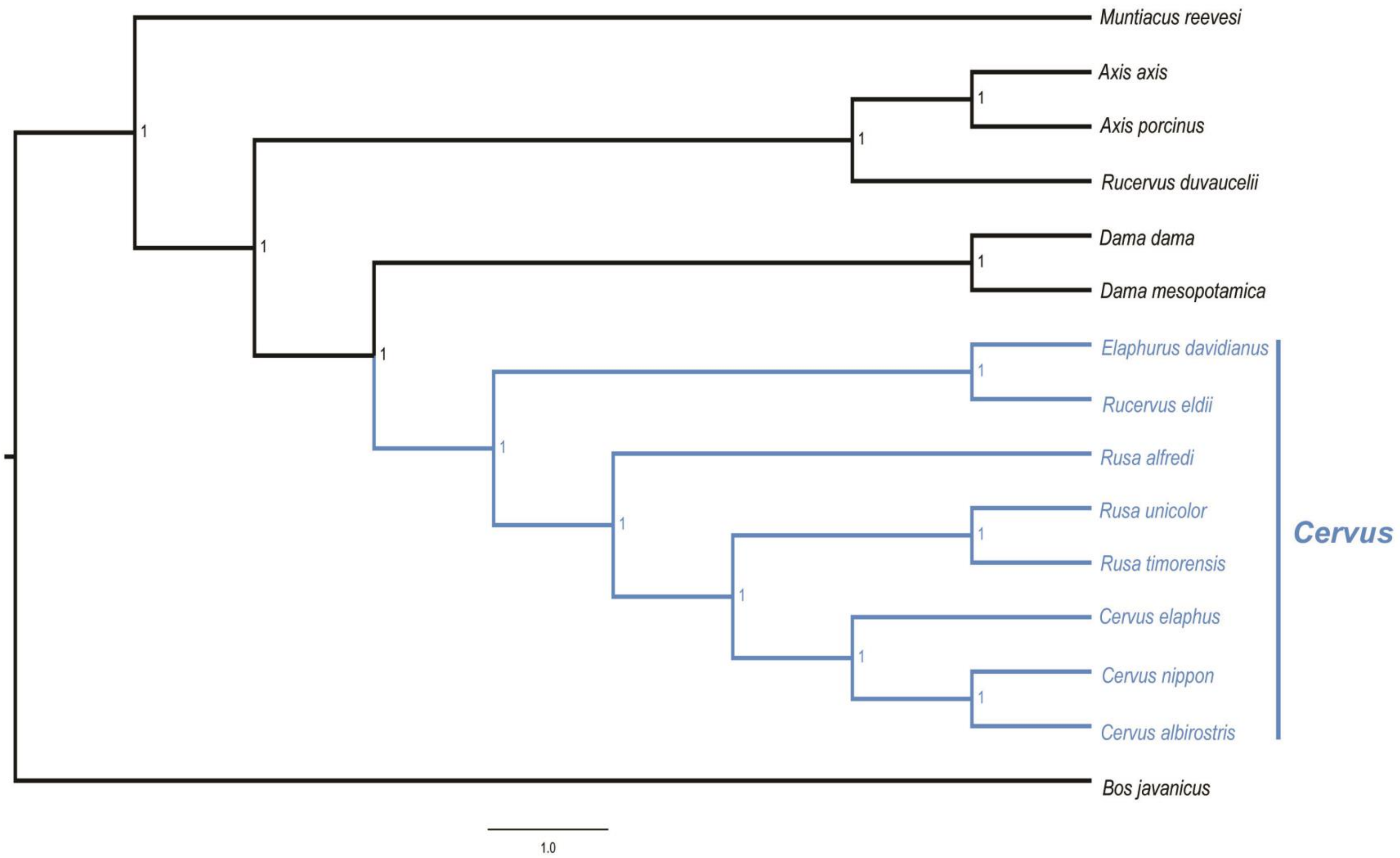

Figure S3. Phylogenetic tree of the tribe *Cervini* obtained using Bayesian analysis of mitochondrial genomes. Bayesian Posterior Probability (BPP) values are given at the respective nodes. Blue line represents the species proposed to be classified in the genus *Cervus*.

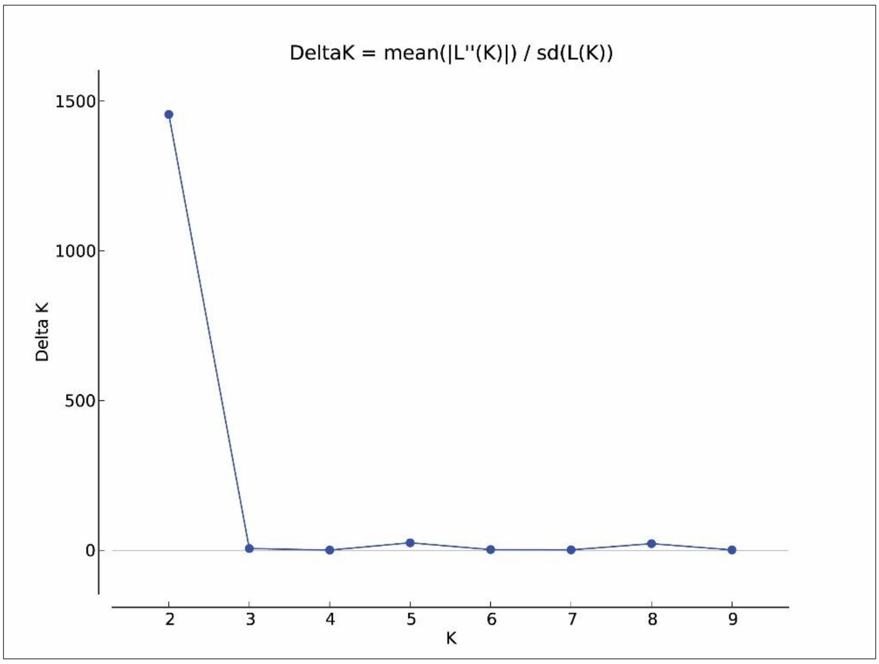

Figure S4. Plot showing the number of optimum genetic clusters in captive and wild populations of *R. e. eldii* obtained using program STRUCTURE.

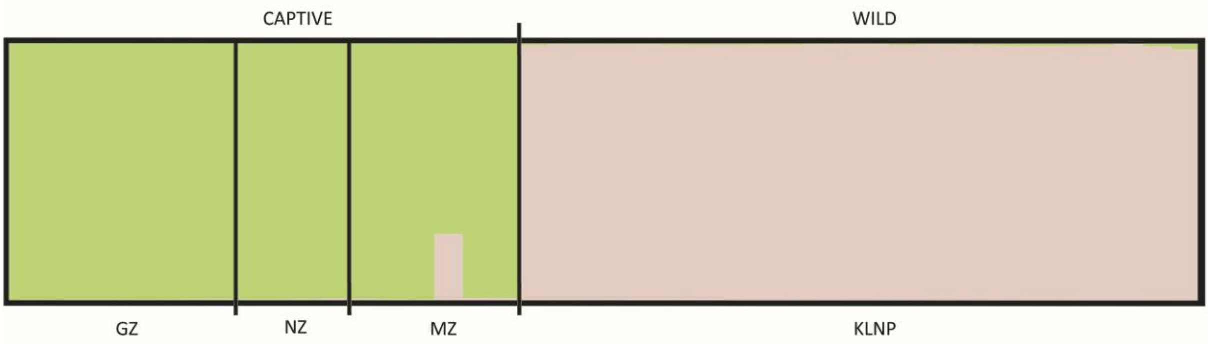

Figure S5. Population genetic structure of captive and wild populations of *R. e. eldii* inferred from 19 microsatellite loci. The captive populations are abbreviated as GZ=Assam State Zoo, NZ=National Zoological Park, MZ=Manipur Zoological Garden, and the wild population as KLNP=Keibul Lamjao National Park.

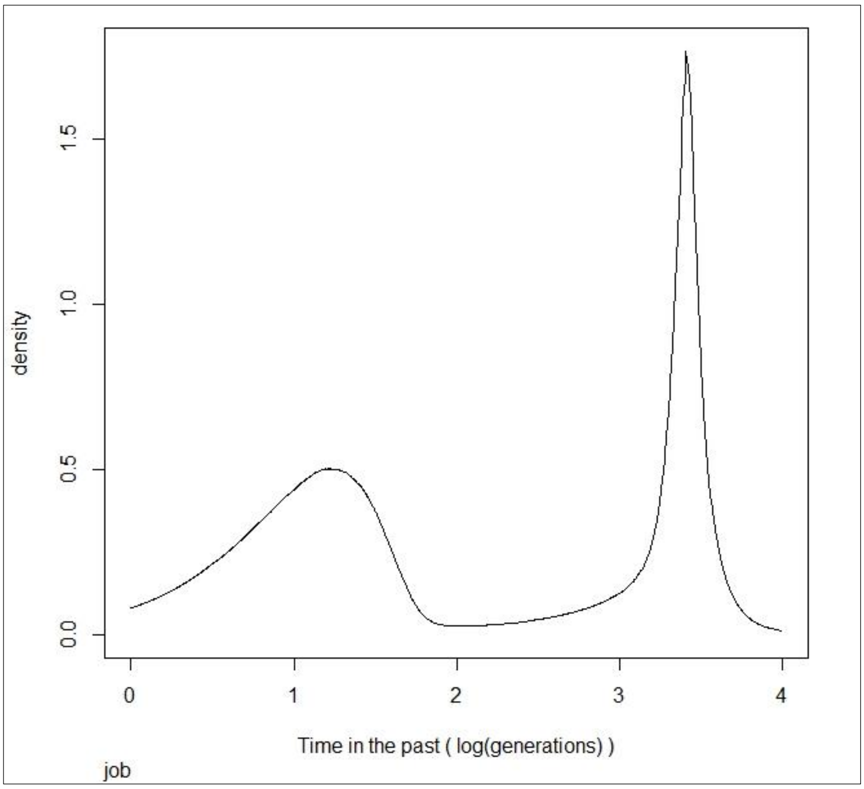

Figure S6. Posterior distribution of the time to most recent common ancestor (T_{MRCA}).

Additional references

[104] Pang, J., Hoelzel, A. R., Song, Y., Zeng, Z. & Zhang, Y. Lack of mtDNA control region variation in Hainan Eld's deer: Consequence of a recent population bottleneck?. *Cons. Genet*. **4**, 109–112 (2003).

[105] Randi, E., Mucci, N., Claro-Hergueta, F., Bonnet, A. & Douzery, E. J. P. A mitochondrial DNA control region phylogeny of the Cervinae: speciation in Cervus and implications for conservation. *Ani. Cons*. **4**, 1–11 (2001).

[106] Liu, H., Dong, Y., Xing, X. & Yang, F. Characterization of the complete mitochondrial genome of Rusa unicolor hainana (Artiodactyla: Cervidae). *Cons. Genet. Res*. **11**, 143–146 (2018).
